# Glossopharyngeal Nerve Block versus Lidocaine Spray to Improve Tolerance in Upper Gastrointestinal Endoscopy

**DOI:** 10.1155/2013/264509

**Published:** 2013-03-05

**Authors:** Moisés Ortega Ramírez, Benigno Linares Segovia, Marco Antonio García Cuevas, Jorge Luis Sánchez Romero, Illich Botello Buenrostro, Norma Amador Licona, Juan Manuel Guízar Mendoza, Jesús Francisco Guerrero Romero, Víctor Manuel Vázquez Zárate

**Affiliations:** ^1^PEMEX Regional Hospital Salamanca, Subdirección de Servicios de Salud, Gerencia de Servicios Médicos. Av. Tampico 910 Col. Bellavista, 36730 Salamanca, Guanajuato, Mexico; ^2^Division of Health Sciences, Department of Medicine and Nutrition, Campus Leon, University of Guanajuato, 20 de Enero No. 929, Colonia Obregón, 37320 León, Guanajuato, Mexico; ^3^Instituto Mexicano del Seguro Social, Director of Education and Research, UMAE HE 1, Paseo de los Insurgentes SN, Colonia Paraísos, 37150 León, Guanajuato, Mexico; ^4^Universidad De LaSalle Bajío, School of Dentistry, Av. Universidad 602, Col. Lomas del Campestre, 37150 León, Guanajuato, Mexico; ^5^PEMEX Subdirección de Servicios de Salud, Av. Marina Nacional 350 Col. Petroleos Mexicanos, 11311 Distrito Federal, Mexico

## Abstract

*Aim of the Study*. To compare the effect of glossopharyngeal nerve block with topical anesthesia on the tolerance of patients to upper gastrointestinal endoscopy. *Methods*. We performed a clinical trial in one hundred patients undergoing upper gastrointestinal endoscopy. Subjects were randomly assigned to one of the following two groups: (1) treatment with bilateral glossopharyngeal nerve block (GFNB) and intravenous midazolam or (2) treatment with topical anesthetic (TASS) and intravenous midazolam. We evaluated sedation, tolerance to the procedure, hemodynamic stability, and adverse symptoms. *Results*. We studied 46 men and 54 women, from 17 to 78 years of age. The procedure was reported without discomfort in 48 patients (88%) in the GFNB group and 32 (64%) in the TAAS group; 6 patients (12%) in GFNB group and 18 (36%) in TAAS group reported the procedure as little discomfort (*χ* ^2^ = 3.95, *P* = 0.04). There was no difference in frequency of nausea (4% in both groups) and retching, 4% versus 8% for GFNB and TASS group, respectively (*P* = 0.55). *Conclusions*. The use of glossopharyngeal nerve block provides greater comfort and tolerance to the patient undergoing upper gastrointestinal endoscopy. It also reduces the need for sedation.

## 1. Introduction

Gastrointestinal endoscopy is usually a minimally invasive procedure and is performed more frequently outside the surgical area [1]. Although the upper gastrointestinal endoscopy or esophagogastroduodenoscopy (EGD) is fairly safe, there is a risk of complications including bleeding, perforation, infection, and adverse drug reactions [2–5]. 

The anesthetic management most frequently used in patients undergoing upper gastrointestinal endoscopy is the combination of topical anesthetics and sedation with benzodiazepines. However, the sedation has been associated with pulmonary and cardiovascular complications. Patients with advanced age and those with cardiopulmonary disease may carry an increased risk for the procedure especially when high doses of intravenous sedatives are used [2, 6–8]. This has led to search for modes of anesthesia that carry less complication rates and, at the same time, provide satisfaction for both patient and endoscopist [9–16]. Few studies have used different forms of topical anesthesia, including spray, lollipop, and inhaler with mixed results. Some of these topical agents still carried a risk of retching, vomiting, and apnea [17–21].

The glossopharyngeal nerve block could be an effective anesthetic alternative. Because the glossopharyngeal nerve is quite superficial, it achieved an adequate block of the posterior third of the tongue and the vallecula. The complications of this technique are rare and less severe. Because of the low doses of local anesthetic used, it is unlikely to have systemic manifestations secondary to intravascular deposition [22, 23].

## 2. Materials and Methods

### 2.1. Patients

We included patients undergoing diagnostic EGD for various indications at the American Society for Gastrointestinal Endoscopy (ASGE). The study was approved by the local ethical committee at the PEMEX Hospital and by the Center of Bioethics of University of Guanajuato.

The sample size was determined with the formula of proportions with a confiability of 95%, 90% of power and considering a reduction of at least 20% in the frequency of pain in the study group.

### 2.2. Study Design

We performed a double-blind clinical trial in patients between 17 and 78 years old undergoing diagnostic EGD at the Endoscopy Department of PEMEX Regional Hospital, in Salamanca, Mexico. In all cases they showed ASA I or II.

Patients were randomly assigned to one of two study groups: treatment with bilateral glossopharyngeal nerve block (GFNB) or treatment with topical anesthetic (TAAS). We evaluated sedation, tolerance to the procedure, hemodynamic stability, duration of the procedure, and adverse symptoms. The decision to administer intravenous sedation during the procedure (in all cases midazolam) was taken by the anesthesiologist depending on the patient's tolerance and the presence of signs of discomfort, like excessive gag, retching, or restlessness. 

GFNB group was treated with midazolam 0.02 mg/kg intravenously and between 2 and 3 minutes later the glossopharyngeal nerve block was performed with 60 mg of lidocaine 2%. Block of the lingual branch of the glossopharyngeal nerve was performed using 60 mg of lidocaine 2% in a syringe with a needle 22 × 32 mm. The tongue was retracted medially, and the needle was inserted under the mucosa at the base of the pillar, 0.5 cm lateral to the base of the tongue 30 mg lidocaine were injected in each side. Blockade was verified by gag reflex provocation after 3 minutes of latency, and then the endoscopic procedure began. 

TAAS group was treated with 30 mg of lidocaine spray, it was administered using the same technique in 3 consecutive 30 s intervals, each consisting of 10 sprays (10 mg/dose) of Xylocaine Pump Spray 10% (AstraZeneca) and 2-3 minutes later, midazolam 0.1 mg/kg intravenously was administered to a maximum dose of 0.2 mg/kg. All the patients had intravenous lines inserted and their vital signs (blood pressure, heart rate, respiratory rate) and pulse oximetry were continuously monitored during the procedure.

The endoscopist and the researcher who evaluated endpoints were blinded to the randomization. The endoscope used in the procedures was the EVIS EXERA-CV160 (Olympus Optical, Tokyo, Japan). Data were collected from the patient's clinical record, including demographic variables such as age and gender in addition to the following clinical parameters: past medical and surgical history, medications, allergies, and history of previous endoscopy. The anxiety level of patients was evaluated according to a scale of 0 to 5 (1 = no anxiety to 5 = extreme anxiety).

### 2.3. Endoscopist's Assessments

After the administration of the local anesthetics, the endoscopist rated the gag reflex based on a scale from 1 to 5 (1 = absent to 5 = strong). After the procedure, the endoscopist determined the ease of the procedure based on a scale from 1 to 5 (1 = easy to 5 = difficult). Finally, the amount of intravenous sedation given was recorded.

### 2.4. Patients' Assessments

After the procedure was concluded, patients were monitored in the recovery room. A questionnaire was filled in by the participants to determine tolerance to the procedure based on a scale from 1 to 5 (1 = no discomfort to 5 = intolerable). Also, symptoms, such as retching, nausea, vomiting, abdominal pain, dyspnea, and cough, during and until 1 hour after (sore throat, nausea, vomiting, abdominal pain, dyspnea, cough) the procedure were recorded.

### 2.5. Statistical Analysis

Results are expressed as mean ± SD or as median (95% CI) according to variables' distribution. The nonparametric Mann-Whitney *U* test was used to compare ordinal variables. The *χ* ^2^ test was utilized to compare categorical variables between the 2 groups. Continuous variables were assessed with an independent sample *t*-test. A *P* value < 0.05 was considered to be significant.

## 3. Results

We studied 100 subjects, fifty subjects per group (46 males and 54 females) with a mean age of 45.3 ± 17.3 years. No difference was found at baseline in gender, BMI, smoking, or previous EGD between groups; however, diastolic blood pressure was higher in the group of GFNB (Table 1). Before the endoscopic procedure, there was no significant difference in anxiety level between the two groups; the median anxiety scores were 2 (95% CI: 2-3) for subjects in GFNB and TASS. The TASS group had significantly stronger gag reflex than the GNFB (*P* = 0.0002) with respective median scores of 4 (95% CI: 3-4) and 2 (95% CI: 1-2).

### 3.1. Intravenous Sedation Use

Intravenous sedation was administered more frequently in the TASS than in the GFNB (92% versus 24%, *χ* ^2^ = 24.3, *P* = 0.0001). The amount of midazolam administered was lower in the GFNB compared to the TASS, 1.34 ± 0.17 mg versus 14.5 ± 2.2 mg (*P* = 0.0001). After the procedure, heart rate, oxygen saturation, and blood pressure were higher in the group GFNB, while the time required to Ramsay II and waking was lower (Table 2). 

### 3.2. Procedure's Tolerance

Patients in the GFNB showed a higher tolerance to the procedure with a median tolerability score of 1 (95% CI: 1-3) as compared to 3 (95% CI: 2-3) in the TASS group. The procedure was reported without discomfort in 44 patients (88%) in GFNB group and 32 (64%) in TAAS group; 6 patients (12%) in GFNB group and 18 (36%) in TASS group reported the procedure as little discomfort (*χ* ^2^ = 3.95, *P* = 0.04). No patient reported much or intolerable discomfort. 

Side effects during and after the procedure were similar in both groups except for pain. Four percent of patients in the GFNB group and 16% of them in the TASS group reported moderate pain during the procedure (*P* = 0.03) with median visual analog scale scores of 1 (95% CI: 1-2) in both groups. There was no difference in frequency of nausea (4% in both groups) and retching 4% versus 8% for groups GFNB and TASS, respectively, (*P* = 0.55).

### 3.3. Endoscopists' Evaluation

The endoscopist's assessment of the degree of procedure difficulty showed that the procedures were significantly easier to perform in the GFNB than in the TASS group. The procedures were reported as moderately difficult in 8% patients in group GFNB and 35% in group TASS (*P* = 0.04), with median difficulty scores of 1 (95% CI: 1-2) and 3 (95% CI: 2–4), respectively. None of the procedures was aborted due to complications, excessive agitation, or major patient discomfort.

## 4. Discussion

The goal of the anesthesiologist in upper gastrointestinal endoscopy is to facilitate the work of the endoscopist giving greater patient safety. Some authors have recommended upper gastrointestinal endoscopy under topical anesthesia without sedation, while others prefer sedation for this procedure because of their anxiolytic, sedative, to produce anterograde amnesia [24–26]. This study was designed under the premise that the glossopharyngeal nerve blocks with minimal sedation would provide fewer side effects and rapid recovery to the patient.

The hemodynamic variables of patients treated with GFNB were better than those in the TASS group, suggesting that GFNB could represent less risk to hypoxemia and other complications that have been reported in patients under local anesthesia and sedation with midazolam [16–19]. Furthermore, our study shows that GFNB effectively suppresses the gag reflex, significantly increases the patient tolerability, and improves endoscopist satisfaction during the procedure.

The need for sedation was greater in the TASS group, while the dose of midazolam required in GFNB group was lower than in the TASS group. 

The procedure duration was 30 minutes in both groups. After the procedure, the GFNB group did not require postoperative observation because of the dose of midazolam for conscious sedation used. However, the TASS group required a postoperative observation for 30 to 45 minutes because of the persistence of sedation. The dose of midazolam used in GFNB group was minimal, so the results cannot be attributed to deficiency in judgment caused by the sedatives used.

This study presents evidence that the use of glossopharyngeal nerve block was an effective way of local anesthesia in upper gastrointestinal endoscopy. The results show that the glossopharyngeal nerve block can be a promising anesthetic technique when it comes to reducing the use of intravenous sedation and potential complications. As a limitation of this technique, glossopharyngeal nerve block during routine diagnostic upper GI endoscopy could be expensive and requires the presence of an anesthetist. However, it may be a promising modality during upper gastrointestinal endoscopy, especially in elderly patients or patients with comorbidities. So it is necessary to compare in future studies this technique in patients with higher ASA score.

## 5. Conclusions

The use of glossopharyngeal nerve block provides greater comfort and tolerance in patients ASA I-II undergoing upper gastrointestinal endoscopy. It facilitates the work of the endoscopist and reduces the need for sedation.

## Figures and Tables

**Table 1:** Baseline characteristics of study groups.

Variable	GFNB *n* = 50	TASS *n* = 50	*P*
Gender (M/F)	22/28	24/26	0.56
Age (years)	47 (29–61)	47 (41–52)	0.77
BMI (kg/m^2^)	25.9 ± 3.7	27.6 ± 2.3	0.14
Heart rate (beats/min)	91.4 ± 16.0	97.8 ± 9.6	0.18
SBP (mmHg)	127.5 ± 8.5	125.3 ± 7.7	0.44
DBP (mmHg)	86.6 ± 7.3	82.1 ± 5.0	0.04
Smoking n (%)	10 (25)	14 (28)	0.50
Previous EGD n (%)	4 (8)	3 (6)	0.68

GNFB: glossopharyngeal nerve block group; TASS: topical anesthetic group; M: male; F: female; BMI: body mass index; SBP: systolic blood pressure; DBP: diastolic blood pressure; EGD: esophagogastroduodenoscopy.

**Table 2:** Hemodynamic variables during upper gastrointestinal endoscopy in relation to anesthetic.

Variable	GFNB *n* = 50	TASS *n* = 50	*P*
Heart rate (beats/min)	76.3 ± 7	70.9 ± 8	0.01
Oxygen saturation (%)	96.9 ± 1.6	91.2 ± 3.5	0.0001
SBP (mmHg)	121.5 ± 12.6	113.5 ± 9.7	0.01
DBP (mmHg)	74.2 ± 9.9	66.8 ± 8.1	0.01
MBP (mmHg)	89.9 ± 10.3	82.3 ± 8.2	0.006
Time for Ramsay II (min)	11.8 ± 4.3	21.8 ± 7.0	0.0001
Time to wake up (min)	10.8 ± 4.3	32.8 ± 8.0	0.0001

GNFB: glossopharyngeal nerve block group; TASS: topical anesthetic group; SBP: systolic blood pressure; DBP: diastolic blood pressure; MBP: mean blood pressure.
